# Myocardial Revascularization in New York State: Variations in the PCI-to-CABG Ratio and Their Implications

**DOI:** 10.1161/JAHA.112.001446

**Published:** 2012-04-24

**Authors:** Wilson Ko, Robert Tranbaugh, Jonathan D. Marmur, Phyllis G. Supino, Jeffrey S. Borer

**Affiliations:** Division of Cardiac Surgery, Beth Israel Medical Center, New York, NY (W.K., R.T.); New York Medical College, Vahalla, NY (W.K.); Albert Einstein School of Medicine, Bronx, NY (R.T.); Division of Cardiology, Department of Medicine, State University of New York-Downstate Medical Center, Brooklyn, NY (P.G.S., J.S.B.)

**Keywords:** CABG, myocardial revascularization, PCI, PCI-CABG ratio

## Abstract

**Background:**

During the past 2 decades, percutaneous coronary intervention (PCI) has increased dramatically compared with coronary artery bypass grafting (CABG) for patients with coronary artery disease. However, although the evidence available to all practitioners is similar, the relative distribution of PCI and CABG appears to differ among hospitals and regions.

**Methods and Results:**

We reviewed the published data from the mandatory New York State Department of Health annual cardiac procedure reports issued from 1994 through 2008 to define trends in PCI and CABG utilization in New York and to compare the PCI/CABG ratios in the metropolitan area to the remainder of the State. During this 15-year interval, the procedure volume changes for CABG, for all cardiac surgeries, for non-CABG cardiac surgeries, and for PCI for New York State were −40%, −20%, +17.5%, and +253%, respectively; for the Manhattan programs, the changes were similar as follows: −61%, −23%, +14%, and +284%. The average PCI/CABG ratio in New York State increased from 1.12 in 1994 to 5.14 in 2008; however, in Manhattan, the average PCI/CABG ratio increased from 1.19 to 8.04 (2008 range: 3.78 to 16.2). The 2008 PCI/CABG ratios of the Manhattan programs were higher than the ratios for New York City programs outside Manhattan, in Long Island, in the northern counties contiguous to New York City, and in the rest of New York State; their averages were 5.84, 5.38, 3.31, and 3.24, respectively. In Manhattan, a patient had a 56% greater chance of receiving PCI than CABG as compared with the rest of New York State; in one Manhattan program, the likelihood was 215% higher.

**Conclusions:**

There are substantial regional and statewide differences in the utilization of PCI versus CABG among cardiac centers in New York, possibly related to patient characteristics, physician biases, and hospital culture. Understanding these disparities may facilitate the selection of the most appropriate, effective, and evidence-based revascularization strategy. **(*J Am Heart Assoc*. 2012;1:e001446 doi: 10.1161/JAHA.112.001446.)**

## Introduction

During the past 2 decades, a dramatic alteration has occurred throughout the world in the application of percutaneous coronary intervention (PCI) and coronary artery bypass grafting (CABG) as therapies for coronary artery disease (CAD). However, while the data underlying use of these procedures are available to all practitioners, the relative distribution of PCI and CABG appears to differ markedly among regions. The magnitude of this difference has not been fully elucidated. Perhaps more importantly, the basis for the difference, which may relate to patient characteristics, physician biases, and other factors, is not well understood.

From 1994 to 2008, the New York State Department of Health (DOH) Cardiac Surgery Advisory Committee (CSAC) published 15 annual reports on adult cardiac surgery entitled *Adult Cardiac Surgery in New York State* and 11 annual reports on adult PCI entitled *Percutaneous Coronary Intervention (Angioplasty) in New York State*.^1^ These tabulations, which are continuing, are generated from data mandated for submission on a quarter-year basis from every New York State program as a condition of continuing DOH accreditation “certificate of need” for performing diagnostic catheterization, PCI, and cardiac surgery.

CSAC has published studies from targeted analyses of selected data from these reports, including comparative data on CABG and PCI in patients with advanced CAD.^2^ It is unclear whether these extensive reports have had any impact on defining acceptable indications for CABG versus PCI “standards of care.” However, this large database, describing the current state of medical practices in New York, may impact importantly on the establishment of benchmarks, public policy develop-ment, allocation of resources, and business development.

Manhattan, one of 5 boroughs (counties) of New York City, boasts a particularly high concentration of medical schools and teaching hospitals and, therefore, of physicians associated with these “academic” institutions. Manhattan's cardiac programs, with their robust reputations for quality of service that attract patients from a large metropolitan area, have contributed importantly to current standards in cardiac care. However, the demography of Manhattan, the expectations of its population, and the attitudes and therapeutic patterns of physicians at academic medical centers may not mirror those of the remainder of New York City, of its neighboring counties, or of New York State as a whole. Thus, as profound changes occur in the application of PCI and the relation of PCI and CABG use, it is of interest to assess the response of the Manhattan programs and to compare these with patterns elsewhere among the entities for which DOH CSAC data are reported. From this assessment, it may be possible to draw useful inferences about the drivers of the changing deployment of these procedures. Particularly, this analysis seeks to define the PCI/CABG ratio performed among academic programs in Manhattan and to compare this with the ratio in the nearby region and to programs in the State as a whole. This ratio may be seen as an index of the practitioners’ attitudes as to the relative appropriateness of the 2 procedures.

## Methods

This study is retrospective and uses all the data publicly available from the 26 reports since 1994 (downloaded from the New York State DOH website) entitled *Adult Cardiac Surgery in New York State* and *Percutaneous Coronary Intervention (PCI) in New York State*. PCI reports were published for 1995, 1997, and 2000, and annually thereafter. Surgery volumes and associated mortality rates are reported annually for each program. The reports include data from only “non-Federal” hospitals because the Veterans Affairs hospitals are not under the State's jurisdiction. Although the data of these patients are not known to the authors of this report, the size of this subset is relatively small and should not impact interpretation of the analysis presented in this study. In addition, New York State does not tally data related to out-of-state residents who have surgeries in New York. Because of geographical proximity, patients from New Jersey, Pennsylvania, Connecticut, and other states and even other countries might add to the totals reported by the DOH CSAC but are not represented. However, it seems unlikely that these numbers are sufficient to materially alter conclusions from this study.

In the interval from 1994 through 2008 there were 9 programs in Manhattan (Presbyterian Hospital [now New York-Presbyterian Columbia University Medical Center], New York Hospital [now New York-Presbyterian Weill Cornell Medical Center], New York University Langone Hospital, Mount Sinai Hospital, Lenox Hill Hospital, Beth Israel Hospital, St. Vincent's Hospital, St. Luke's Hospital, and Bellevue Hospital). There were 7 programs elsewhere in New York City: 2 programs in the Bronx (Montefiore-Moses, Montefiore-Weiler), 3 programs in Brooklyn (University Hospital of Brooklyn, Maimonides Hospital, and New York Methodist Hospital), 1 program in Queens (New York Hospital-Queens), and 1 program in Staten Island (Staten Island University Hospital). Outside New York City but in contiguous and other nearby counties, there were 5 programs in Long Island (North Shore Hospital, Long Island Jewish Hospital, Winthrop University Hospital, University Hospital of Stony Brook, and St. Francis Hospital), and 3 programs in the nearby counties north of New York City (Westchester, Rockland, and Dutchess) that were certified to perform cardiac surgery and PCI (Westchester County Hospital, Good Samaritan-Suffern, Vassar Brothers). These 24 programs are located in the areas that refer most patients to Manhattan academic programs. In the recent years, 2 programs in Queens (Jamaica Hospital, Elmhurst Hospital), 2 programs in Brooklyn (Brookdale Hospital, Long Island College Hospital), and 4 programs in Long Island (Southside Hospital, Good Samaritan Hospital-West Islip, Huntington Hospital, St. Catherine of Siena) have been approved to perform PCI without an on-site cardiac surgery program. PCIs from these nonsurgical programs were included in analyses for each of the relevant regions. In the remainder of New York State, there were 16 other surgical programs and 21 other PCI programs as of the latest report.

### Statistical Analysis

The hospital procedure-related, hospital discharge, and follow-up data used in this study were reported under legal mandate of New York State and, by law, included all residents of the State in non-Federal hospitals who underwent the relevant cardiac surgical and PCI procedures. Therefore, these data represent all those available in the total population of patients who underwent procedures each year. Because the data include *all* observations in the entire population of interest and not a sample drawn from a larger population of observations, the use of inferential statistical tests that assess the probability “*P*” of sample statistics are not appropriate for this analysis. (For example, if one is interested in whether or not the average PCI/CABG ratio for all Manhattan hospitals is different from the average PCI/CABG ratio for all other hospitals in New York City, direct comparison of the ratios is all that is required; no inferential statistical test would be relevant.) The determination as to whether the observed differences among subsets of the population are clinically important must be based on a nonstatistical “clinical” judgment. Therefore, no *P* values are computed for comparisons or trends. Instead, descriptive summaries are provided through tables and graphics showing the observed differences and trends. Because the entire relevant population is presented, rather than a sample, interpretation of these data as to differences and trends can and must be made directly from the reported data without interposition of statistical tests of significance.

## Results

**Figure 1.**
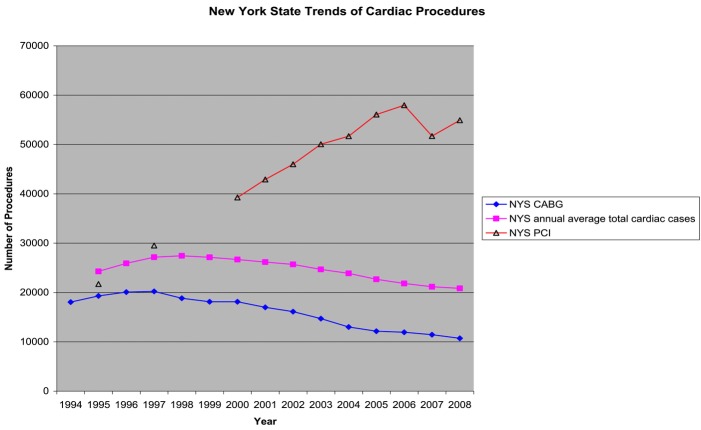
New York State trends of cardiac procedures. Annual average total is the calculated averages from the 3-year running totals published in each report.

**Figure 2.**
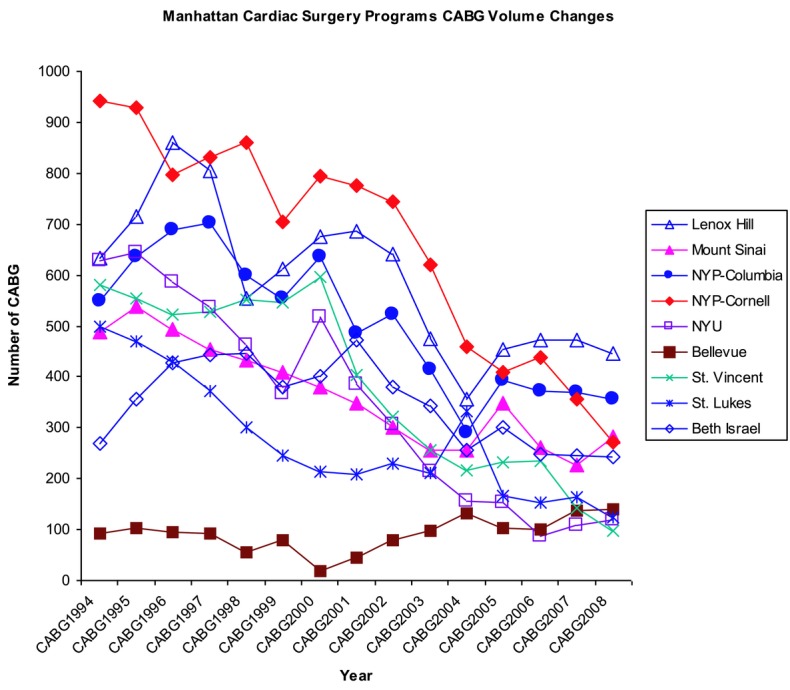
Manhattan Cardiac Surgery Programs CABG volume changes. NYP indicates New York Presbyterian; NYU, New York University.

**Figure 3.**
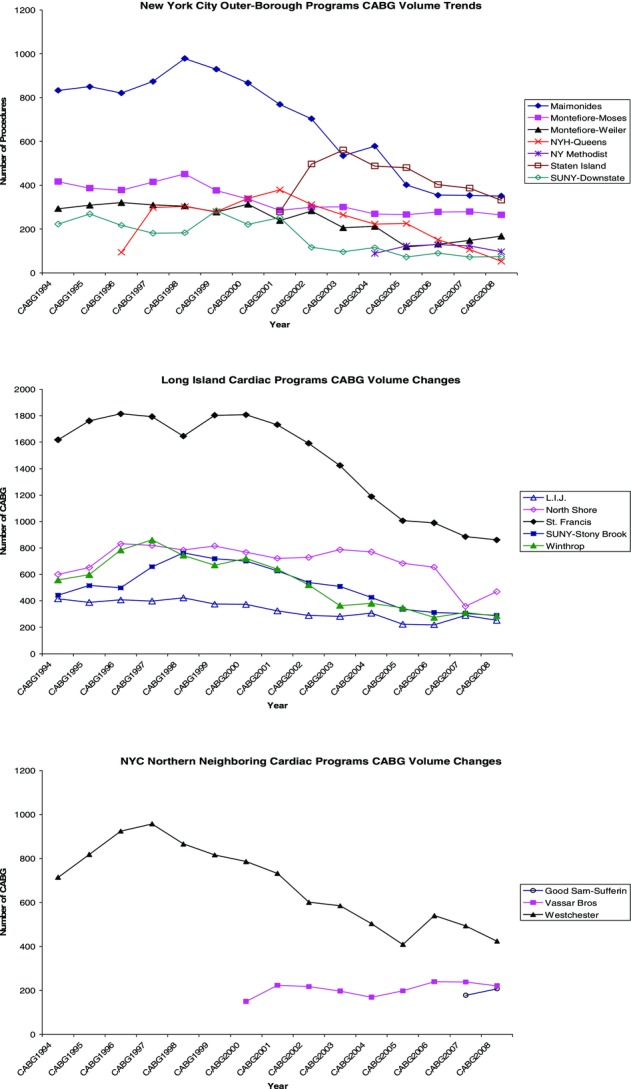
New York City outer boroughs, Long Island, and New York City neighboring northern counties Cardiac Surgery Programs CABG volume changes. NYC indicates New York City; NYH, New York Hospital; SUNY, State University of New York; LIJ, Long Island Jewish; Sam, Samaritan; and Bros, Brothers.

In the reporting period from 1994 to 2008, the New York State CABG case volume decreased by 40%, and 47% compared with the peak year of CABG frequency in 1997 (Figure 1). In contrast, PCI volume increased 267% in 2008 compared with 1995 (Figure 1). The year-to-year changes in CABG volume for the Manhattan programs, New York City outer-borough programs, Long Island programs, and the neighboring upstate programs are illustrated in Figures 2 and 3. All Manhattan programs except Bellevue sustained significant CABG volume reduction in the past decade. The percentage fall in CABG volume (Table 1) for the Manhattan programs (61%) in this interval was higher when compared with the New York City outer-borough programs (43%), Long Island programs (46%), or to the remainder of the New York upstate programs (33%; 2008 CABG volume included the cases done in the new programs opened in the recent years). Interprogram variation in Manhattan was relatively large, ranging from 46% to 85% (Table 1). Factors potentially confounding this variation, among others, include the loss of some volume to new programs as they were established during the 15-year interval, although these tended to affect existing programs that were specifically allied with centers in which the new units were established; recruitment of surgeons from one program to another or loss of cardiologists and surgeons from one hospital to another; the decision by the New York City Health and Hospitals Corporation to mandate that all invasive procedures in Health and Hospitals Corporation hospitals must go to Bellevue to minimize movement of funds out of the Health and Hospitals Corporation system; and, perhaps, the development of the reputation of one program or another as a “reference center of excellence” to which patients might be referred for a particular procedure but not for others, inflating that arm of the ratio for that institution. However, despite these confounders, the volume reduction was widespread and, by 2008, all programs, even those newly established during the reporting interval, had lost volume from their peak years, with the exception of Bellevue, which is the designated PCI and surgical center for all the Health and Hospitals Corporation hospitals. Moreover, the reduction observed in Manhattan was not attributable to increases in the other boroughs of New York City or in nearby counties: All these programs lost CABG volume, on average proportionally similar to the losses in Manhattan (Table 1).

**Table 1.** Percent Drop in CABG Volumes Among Manhattan Programs Versus New York City Outer-Borough Programs and Long Island Programs

	Peak Year CABG Volume	2008 CABG Volume	Percent Change
**Manhattan programs**			

Lenox Hill	860	445	−48%

Mount Sinai	537	282	−47%

Columbia	702	357	−49%

Cornell	942	273	−71%

NYU	645	118	−82%

Bellevue	103	140	+36%

St. Vincent	580	97	−85%

St. Luke's	500	123	−75%

Beth Israel	446	243	−46%

**Manhattan total**	5315	2078	−61%

**New York City outer-borough programs**			

**Brooklyn**			

Maimonides	979	351	−64%

SUNY-Downstate	284	74	−74%

NY Methodist	129	97	−25%

**Queens**			

NYH-Queens	379	53	−86%

**Bronx**			

Montefiore-Moses	451	265	−41%

Montefiore-Weiler	321	168	−47%

**Staten Island**			

Staten Island University	561	332	−41%

**Brooklyn/Queens**			

**Bronx/Staten Island total**	3104	1340	−43%

**Long Island programs**			

St. Francis	1814	861	−53%

North Shore	831	469	−43.5%

LIJ	423	253	−40%

Winthrop	861	286	−67%

SUNY-Stony Brook	762	290	−62%

**Long Island total**	4691	2159	−46%

LIJ indicates Long Island Jewish; NY, New York; NYH, New York Hospital; NYU, New York University; and SUNY, State University of New York.

### PCI/CABG Ratio

**Figure 4.**
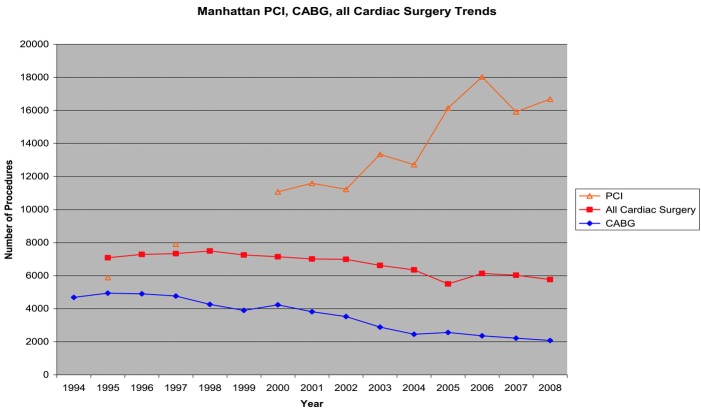
Manhattan PCI and CABG volume trends.

**Figure 5.**
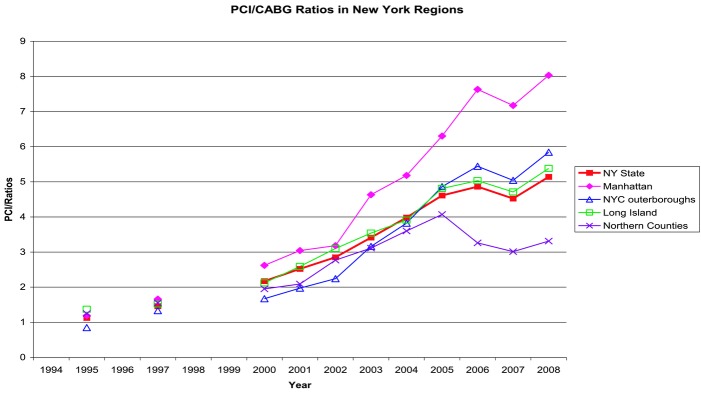
PCI/CABG ratios in and around Manhattan. NYC outer boroughs include Brooklyn, Bronx, Queens, and Staten Island. Long Island includes Nassau and Suffolk counties. Northern counties include Westchester, Rockland, and Dutchess counties north of New York City. NYC indicates New York City.

**Figure 6.**
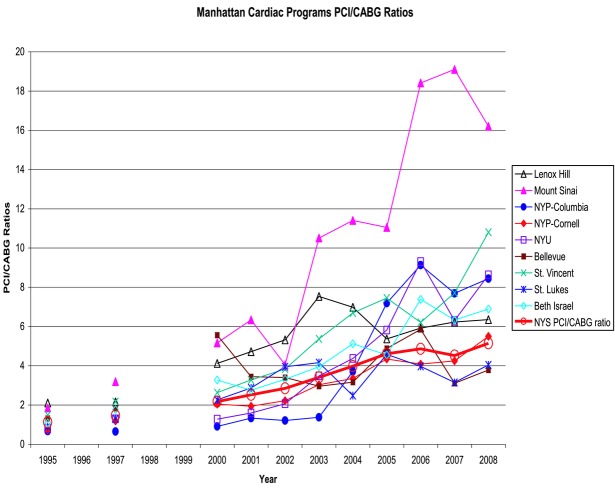
Manhattan Cardiac Programs PCI/CABG ratios. NYP indicates New York Presbyterian; NYS, New York State; and NYU, New York University.

The primary reason for reduction in CABG volume across almost all programs almost certainly was the rapidly progressive substitution of PCI for CABG. The PCI/CABG ratio describes this shift. Alterations in the PCI and CABG volumes in the Manhattan programs as a group (Figure 4) had similar trends as those observed for the New York State (Figure 1). The peak PCI volume occurred in 2006. The PCI volume then decreased slightly and subsequently appeared to reach a plateau. The PCI increase in 2006 compared with 1995 was 267% for New York State (Figure 1), and it was 306% for the Manhattan programs combined (Figure 4). The changes relative to 1995 were slightly lower in 2008. As shown in Figure 5, the average PCI/CABG ratio in New York State increased from 1.12 in 1994 to 5.14 in 2008; in Manhattan, the average PCI/CABG ratio increased from 1.19 to 8.04 (2008 range: 3.78 to 16.2). The 2008 PCI/CABG ratios of the Manhattan programs were higher than the ratios for the programs in New York City outside Manhattan, in Long Island, in the northern counties contiguous to New York City, and in the rest of upstate New York (average ratios 5.84, 5.38, 3.31, and 3.24, respectively; Figure 5). Perhaps even more strikingly, the changes in these ratios varied markedly among the Manhattan programs: In one program, the ratio exceeded 18 (Figure 6). In 2008, 7 programs were above the state average, while 2 were below.

### Total Cardiac Surgery Volume Trends

In New York State, the total cardiac surgery volume decreased 20% by 2008 from its peak in 1998. This was attributable entirely to the 40% drop in CABG volume during this interval (Figure 1) because the total non-CABG cardiac surgeries increased by 17.5%, from 8607 in 1998 to 10 188 in 2008.

**Figure 7.**
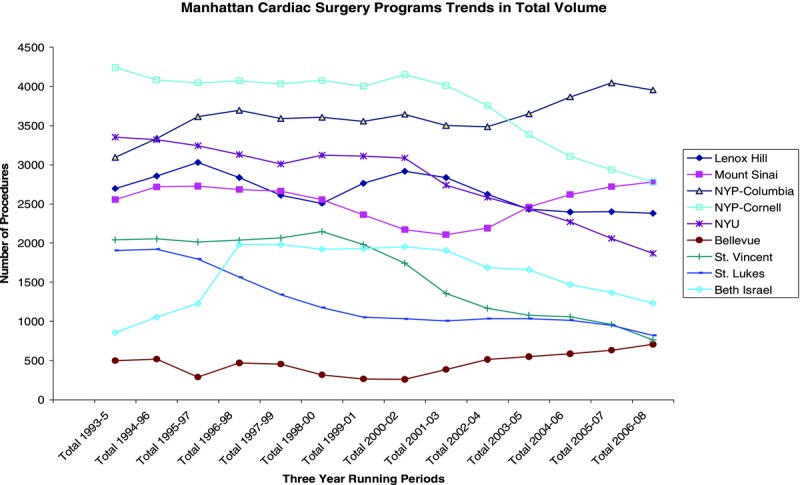
Manhattan Cardiac Surgery Programs trends in total case volumes. Number of procedures are 3-year running totals. NYP indicates New York Presbyterian; NYU, New York University.

In Manhattan, total cardiac surgery volume fell 23% during the same interval (Table 2), while non-CABG cardiac surgery increased 14% (3225 to 3685). However, as for CABG, the magnitude of reduction in total volume varied widely among the programs (Figure 7). The CABG volume in Manhattan at the peak of 1995 accounted for 70% of the total adult cardiac cases (4942 of the 7084 cases); by 2008, CABG accounted for only 36% (2078 out of 5763 total adult cases, Table 2).

**Table 2.** Maximum 2008 Changes in Total Cardiac Case Volumes in Manhattan

Manhattan Programs	Peak CABG Percentage*	2008 CABG Percentage†	2008 Maximum Change in Total Cardiac Surgery Cases‡
Bellevue	62%	59%	+36%

Mount Sinai	63%	30%	+2%

NYP-Columbia	58%	27%	+7%

Lenox Hill	90%	56%	−22%

NYP-Cornell	67%	29%	−35%

Beth Israel	67%	59%	−38%

NYU	58%	19%	−44%

St. Luke's	79%	45%	−57%

St. Vincent	85%	38%	−63%

All programs combined	69%	36%	−23%

NYP indicates New York Presbyterian; NYU, New York University.

* The highest annual CABG percentage calculated as the percentage of the total cardiac cases that were CABG in the reporting since 1994.

† Percentage of the total cardiac cases that are CABG in 2008.

‡ Peak changes calculated on the basis of 2008 calculated annual volume compared with the highest volume in the early part of the 15-year reporting period.

#### Mortality Rates

**Figure 8.**
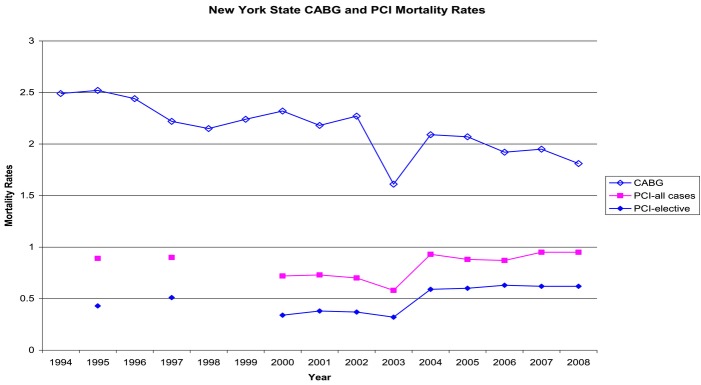
New York State CABG and PCI mortality rates.

In the 15 years, from 1994 through 2008, on which this analysis is focused, the mortality rate for PCI has remained steady just below 1%, while that for CABG has fallen steadily from 2.5% to 1.8% (Figure 8).

## Conclusion

For New York State between 1994 and 2008, cardiac programs demonstrated a marked reduction in CABG surgery, a moderate increase in non-CABG cardiac surgery, and a marked increase in PCI, leading to a dramatic increase in the ratio of PCI to CABG. The change was directionally similar, although the increase in the ratio was significantly higher in Manhattan, with wide variations among the academic programs. The rise in the utilization of PCI during the introduction of drug-eluting stents in the interval between 2002 and 2004 was greatly anticipated at that time. However, the New York State PCI utilization data do not fully support this anticipated result. As can be extrapolated from Figure 1, the rate of increase in PCI in New York State has been linear from 1995 to 2008, and the slope of this linear rise did not appear to have changed when drug-eluting stents were adopted. It is more likely that the introduction of drug-eluting stents was one of many factors, including improving operator skills, experience, catheter, imaging, and other technologic innovations in PCI over the years, that have contributed to maintaining the linear growth of PCI.

Thus, despite the growing and aging population and the steady improvement in the mortality rates of CABG, the utilization of CABG as a treatment option for advanced CAD has declined remarkably. The reasons may include the migration of CABG to PCI and major advances in medical management (application of the statin drugs, as evidenced by the COURAGE trial,^3^ angiotensin-converting enzyme inhibitors [ACE-I] for secondary prevention,^4,5^ progressively more effective “anti-platelet” drugs, enhanced control of hypertension, etc) coupled with increasingly effective diagnostic modalities, supported by increasingly accurate prognostic data, enabling progressively more effective identification of patients at risk who may require particularly intensive management strategies.

The variation in routine practice within Manhattan, and between Manhattan and other parts of New York, stands in marked contrast to the standard of care in other countries and other regions of the United States. This decline in CABG is not observed in Canada, for comparison.^6^ While the PCI/CABG ratio rose from 1.13 in 1994 to only 2.63 in 2005^6^ (an increase rate nearly 2 times less than in New York), the more important difference between these 2 North American countries is that, during this study interval, the CABG volume remained stable, at 70 to 75 per 100 000 people in Canada, while it decreased 60% in Manhattan and 40% in New York State as a whole (despite a 2.17% population growth according to the 2000 and 2010 censuses). The United States population data are available for years 2000 and 2010 (census data are collected once per decade); the present study has CABG volume up to 2008 only. The CABG rate for year 2000 was 18 121/18 976 457, or 96 per 100 000; calculation for the other years of interest is not possible. However, the 2010 census indicated a 2.17% increase in population compared with 2000. The 40% drop in CABG volume in 2008 is highly disproportionate with this New York State population change and strongly suggests that this CABG diminution is not importantly affected by the population change. The New York State population also was stable during the interval when the Canadian data were collected.

The decision to apply PCI versus CABG lies with the gatekeepers, the physicians who perform diagnostic cardiac catheterizations, as well as with the referring physicians and, ultimately, of course, with the patients. The data reveal a disproportionate loss of CABG volume in Manhattan as compared with the remainder of New York City, the nearby suburban counties, and the State as a whole and a disproportionate rise in the utilization of PCI in Manhattan as compared with these other areas. As a result, the PCI/CABG ratio rose disproportionately in Manhattan, as well.

The PCI/CABG ratio is an index of relative resource utilization. It is also an indication of physician and patient attitudes about the appropriate strategies for managing advanced CAD. The most striking aspect of the variation of this ratio over time is the marked difference in Manhattan compared with New York State as a whole and with subregions of the State, including those contiguous or nearby to Manhattan. By 2008, a patient with advanced CAD presenting in Manhattan had a 56% greater chance of receiving PCI than CABG as compared with the rest of New York State (PCI/CABG ratios of 8.04 vs 5.14). In one Manhattan program the likelihood of PCI was dramatically greater (215%) than for the remainder of New York State, or 203% greater than in nearby Long Island, and 177% greater than in the remainder of New York City.

Although much of the published evidence precedes the era of current medical therapies, data available from randomized, controlled trials indicate life-prolonging benefit from CABG in several subpopulations with severe advanced CAD (eg, the Coronary Artery Surgery Study trial^7^). Such data do not yet exist for PCI. In addition, recent evidence from the DOH CSAC reports strongly suggests that CABG effects on long-term survival are superior to those of PCI,^2^ among the 59 314 treated patients with 3-vessel CAD between 1997 and 2000 (CABG had a 36% survival advantage over PCI; relative mortality risk, 0.64, *P*<0.05). Although this report is faulted for being retrospective and not randomized, the data were comprehensive and represented the real-world experience in New York. The introduction of drug-eluting stents has not changed this survival advantage of CABG over PCI.^8^

Similarly, for patients with left-main or triple-vessel CAD, the superiority of CABG for prevention of major adverse cardiac events (including all-cause mortality, myocardial infarction, stroke, and need for repeat revascularization) is apparent as early as the first year after the procedure, as demonstrated in the recently published prospective randomized SYNTAX trial^9^; 3-year data presented in the 2011 Society of Thoracic Surgeons Annual meeting, but yet to be published, showed similar benefits of CABG over PCI.

Recent reports have focused on the reasons for disproportionate use of PCI in certain metropolitan areas of the United States. One study compared the utilization of PCI and CABG for specific indications in the government-sponsored programs in Ontario, Canada, versus that in the New York State. The authors concluded that “the market-oriented financing approach in New York State is associated with markedly higher rates of PCI procedures for both discretionary indications (ie, PCI in non-acute myocardial infarction patients) and emergent indications (ie, primary PCI for myocardial infarction) compared with the Government-funded single-payer system in Ontario,” whereas the utilization of CABG was the same in the 2 locales.^10^

Recent guidelines on myocardial revascularization formulated by the task force of the European Society of Cardiology and European Association for Cardio-Thoracic Surgery have focused on the concept of a “heart team” or a panel of the stakeholders to provide treatment recommendation to these patients.^11^ Recently the “ACCF/AHA Guideline for Coronary Bypass Graft Surgery: A Report of the American College of Cardiology Foundation/American Heart Association Task Force on Practice Guidelines” was published online. This includes a Class I recommendation for a heart-team approach.^12^ This approach was not in use in New York during the years these data were collected. Moreover, there are legitimate reasons to deviate from “guidelines” or from “evidence-based” practices when decisions are made for individual patients and, particularly, when the strength of evidence is importantly deficient, but practitioners and hospitals should document such deviations and account for a pattern of persistent deviation. In a recent study on 19 centers in New York,^13^ Hannan used the American College of Cardiology/American Heart Association guidelines to assess the indication of over 10 000 patients after diagnostic catheterization. The patients were categorized into 3 groups: CABG indicated, PCI indicated, and CABG or PCI indicated. In the PCI-indicated group, 94% of patients underwent PCI. In the CABG-indicated group, only 53% underwent CABG, while 34% underwent PCI. In the CABG- or PCI-indicated group, 93% had PCI. The catheterization laboratory cardiologist was the “final source of recommendation” for 64% of these patients. The findings of this study illustrate the large discrepancy between evidence-based guidelines and real-world practice in New York, suggest the potential utility of the heart-team approach, and question whether a meaningful conflict of interest exists if the decider is the operator of 1 of 2 options. The same bias exists if a patient is referred to a surgeon by the diagnostic cardiologist without the benefit of a consultation by an interventional cardiologist; this is of course an infrequent scenario. The norm in New York is that the decision is made with the patient on the catheterization laboratory table based on the recommendation from the interventional cardiologist. The recent availability of the SYNTAX score^14^ to assess the overall burden of CAD in terms of the number of lesions, location, complexity, etc, may assist the “heart team” to provide a more objective recommendation on aggressive medical management, PCI, and CABG.

The 40% migration of CABG to PCI does not account entirely for the 256% increase in PCI utilization in New York State during the 15-year interval assessed in this study. Despite improved medical management over time, PCI application has increased dramatically in absolute as well as relative terms during the interval. The reasons include PCI application in patients with less than critical disease based on biases about long-term effects that are not yet proven, repeat PCI procedures on the same patient when initial therapy is inadequately durable in its effects, application of PCI to patients who have had CABG and whose grafts have failed or who have developed new native vessel disease and who do not wish to undergo additional surgical procedures, rapid technologic advances in PCI that lead to PCI in patients who would have been referred to surgery in the past, aggressive screening and imaging evaluation of patients with potential CAD for whom the benefit of any mechanical intervention is not demonstrated but for whom the theoretical attractiveness of “opening the artery” is particularly compelling to patient and operator, increasing prevalence of obesity-related type II diabetes mellitus causing CAD and the concerns about diabetes-related complications of surgery (though PCI is relatively ineffective in such patients in comparison to surgery^15^), and increasing awareness of the success and relative safety of PCI in the general public in conjunction with the general fear of the misnamed “open heart” surgery. The less traumatic nature and rapid recovery from PCI compared with surgery are attractive benefits for relatively young patients who wish to return to work and avoid short-term economic detriment, despite potentially greater long-term benefits of CABG.

CABG and PCI each offer important benefits for subgroups of patients with CAD. However, as demonstrated in this review of New York State DOH CSAC data, large disparities have developed in relative PCI/CABG utilization ratios among New York State programs in the recent years. As yet no data exist to inform and optimize determination of the most appropriate application of these procedures for the individual patient. This decision requires consideration of long-term benefits as well as short-term risks and inconvenience and requires that patients be adequately informed about the outcomes that can be expected with either of the 2 modalities. A parallel need is the development of data to fill the large knowledge gaps that limit the accuracy of information that can be provided to patients. Consideration of reducing pressures to complete PCI at the time of diagnostic catheterization, an economic issue, may enable more reasoned decisions by practitioners and patients.

Most recently, intense medical management in patients with stable CAD was found to be equally effective in preventing death, nonfatal myocardial infarction, unplanned revascularization, and persistent angina in comparison to initial PCI in a meta-analysis of several large clinical trials.^16^ Indeed, there is now intense controversy as to whether invasive plus pharmacological management or pharmacological management alone should be the initial strategy for patients with chronic stable CAD.^17^ It is anticipated that this controversy will reduce the utilization of PCI, particularly in patients with relatively lower disease burden. It will be useful to assess whether this anticipated lower overall PCI utilization will further lower the utilization of CABG in patients with high disease burden, perhaps because of further erosion of CABG referral to make up for the loss in PCI volume in cardiology practices.

According to data released by the National Heart, Lung, and Blood Institute, by 2009, cardiovascular mortality rate in the United States had decreased 79% compared with the peak in 1959.^18^ The causes for this dramatic change undoubtedly are multiple, including improvements in diagnostic modalities (imaging, etc), enhanced understanding of disease pathophysiology leading to increasingly effective pharmacological treatments, and the efficacy of CABG and PCI on outcomes that have been demonstrated in certain segments of the CAD-afflicted population. Further mortality rate reduction and other benefits certainly are achievable but will be optimally realized only if we demand more and better data, regularly updated as technological advances continue, about relative effectiveness and appropriate application of available therapies.

## Limitations

(1) Our underlying assumption is that, given the large size of the populations involved and the relatively homogenous training of New York State physicians, the patient factors (coronary disease burden and comorbidities) are relatively homogenous among the different regions and are highly unlikely to account for the striking differences in PCI/CABG ratio identified among the different boroughs of New York City and among the different regions of New York State. These seem far more likely to relate to differences in the physicians’ beliefs and practices grounded in factors like those we have discussed. However, data do not exist to allow objective evaluation of this assumption. (2) The New York State DOH requires each standing catheterization laboratory that performs PCI to have a close affiliation with a standing cardiac surgery program, which always must be in reasonable geographic proximity to the PCI program. Patients are not required to have surgery at the affiliated surgical program. However, it is unlikely that these patients travel far outside of their region for surgery. This study was designed to preclude missing any PCIs performed in the entire state in our calculation and analysis. All the patients from these laboratories would have had surgery at the programs that were included in this study unless they had surgery outside of New York State. Thus, the overall ratio should not be importantly affected by movement from the catheterizing hospital to another NYS hospital for surgery; indeed, because most patients reasonably can be expected to stay within their region, it is unlikely that the regional ratios will be importantly affected by programs that have PCI without CABG. Again, however, we have no objective data with which to evaluate these assumptions. (3) The occurrence of multiple procedures in individual patients was not considered in this analysis. Unfortunately, such data are not available from the New York State CAC Database. However, on the basis of the practice pattern of the authors and of those who have worked with them in some of the larger programs in this city and state, the number of patients who undergo multiple procedures is relatively small compared with the total number of procedures performed in the state. We believe that this factor is unlikely to have had a substantial impact on the analysis. (4) During the interval of this study, the number of interventional cardiologists increased and the number of cardiac surgeons decreased in New York State. However, it is difficult to attribute the changes in the number of operators to the procedure utilization, although it is certainly a very reasonable observation and hypothesis.
